# Feature-Shuffle and Multi-Head Attention-Based Autoencoder for Eliminating Electrode Motion Noise in ECG Applications

**DOI:** 10.3390/s25206322

**Published:** 2025-10-13

**Authors:** Szu-Ting Wang, Wen-Yen Hsu, Shin-Chi Lai, Ming-Hwa Sheu, Chuan-Yu Chang, Shih-Chang Hsia, Szu-Hong Wang

**Affiliations:** 1 Department of Computer Science and Information Engineering, Chaoyang University of Technology, Taichung City, Wufeng 413310, Taiwan; stwang@cyut.edu.tw; 2 Department of Electronic Engineering, National Yunlin University of Science and Technology, Yunlin County, Douliu 64002, Taiwan; xwy1204@gmail.com (W.-Y.H.); sheumh@yuntech.edu.tw (M.-H.S.); hsia@yuntech.edu.tw (S.-C.H.); wangsr@yuntech.edu.tw (S.-H.W.); 3 Department of Automation Engineering and the Smart Machinery and Intelligent Manufacturing Research Center, National Formosa University, Yunlin County, Huwei 632301, Taiwan; 4 Department of Computer Science and Information Engineering, National Yunlin University of Science and Technology, Yunlin County, Douliu 64002, Taiwan; chuanyu@yuntech.edu.tw

**Keywords:** electrode motion artifacts, autoencoder, ECG denoising, multi-head self-attention, transformer

## Abstract

Electrocardiograms (ECGs) are critical for cardiovascular disease diagnosis, but their accuracy is often compromised by electrode motion (EM) artifacts—large, nonstationary distortions caused by patient movement and electrode-skin interface shifts. These artifacts overlap in frequency with genuine cardiac signals, rendering traditional filtering methods ineffective and increasing the risk of false alarms and misdiagnosis, particularly in wearable and ambulatory ECG applications. To address this, we propose the Feature-Shuffle Multi-Head Attention Autoencoder (FMHA-AE), a novel architecture integrating multi-head self-attention (MHSA) and a feature-shuffle mechanism to enhance ECG denoising. MHSA captures long-range temporal and spatial dependencies, while feature shuffling improves representation robustness and generalization. Experimental results show that FMHA-AE achieves an average signal-to-noise ratio (SNR) improvement of 25.34 dB and a percentage root mean square difference (PRD) of 10.29%, outperforming conventional wavelet-based and deep learning baselines. These results confirm the model’s ability to retain critical ECG morphology while effectively removing noise. FMHA-AE demonstrates strong potential for real-time ECG monitoring in mobile and clinical environments. This work contributes an efficient deep learning approach for noise-robust ECG analysis, supporting accurate cardiovascular assessment under motion-prone conditions.

## 1. Introduction

Cardiovascular diseases remain a leading cause of death worldwide, showing a significant increase in the number of fatalities over the last three decades. In 2021, the mortality rate from CVD reached over 19 million globally, up from approximately 12 million in 1990. The early and accurate detection of CVDs is crucial for improving patient outcomes and reducing the burden on healthcare systems [1]. Electrocardiograms (ECGs) are widely used for diagnosing heart-related abnormalities, as they provide a non-invasive and cost-effective way to monitor the heart’s electrical activity. However, the utility of ECG signals is often compromised by various types of noise, which can obscure critical diagnostic information.

The modern lifestyle, characterized by increased nocturnal activity, high work pressure, lack of exercise, and unhealthy dietary habits, has led to an increase in the incidence of chronic diseases, particularly heart diseases, at younger ages. Regular health check-ups have thus become crucial, and ECG serves as an effective, non-invasive tool for monitoring heart function. However, ECG signals are often weak and susceptible to various noise types, such as baseline wander (BW), muscle artifacts (MAs), electrode motion (EM) [2], and power line interference (PLI) [3]. This noise can significantly affect diagnostic accuracy and increase healthcare costs. Traditional signal processing techniques, such as adaptive filtering [4,5,6], have been employed to mitigate noise in ECG signals. Rahman et al. [4] proposed efficient and simplified adaptive noise cancelers for ECG signals, designed for sensor-based remote health monitoring, achieving significant noise reduction while maintaining computational efficiency. Sharma et al. [5] introduced an adaptive filter using the LMS algorithm to effectively reduce noise in ECG signals, demonstrating its efficiency in enhancing signal clarity in real-time applications. Shaddeli et al. [6] extended this concept by incorporating a variable step-size least mean square (LMS) algorithm optimized with evolutionary algorithms, improving adaptability to dynamic noise conditions.

However, when dealing with more complex signal structures or nonlinear dynamic systems, adaptive filtering alone may not sufficiently separate target signals from noise. To address these challenges, advanced signal decomposition techniques such as empirical mode decomposition (EMD) [7] and wavelet transform (WT) [8,9] have been developed to offer more robust solutions for ECG denoising. EMD adaptively decomposes a signal into a series of Intrinsic Mode Functions (IMFs), each representing oscillatory modes at different frequency bands. An integrated EMD adaptive threshold denoising method (IEMD-ATD) [7] has been proposed to improve decomposition quality and stability in ECG processing. This approach combines complete ensemble empirical mode decomposition with adaptive noise (CEEMDAN) to effectively reduce noise in ECG signals. Wavelet transform-based techniques have also shown promise for ECG noise reduction. In contrast to EMD, DWT uses mathematically defined wavelet bases to decompose signals. Through multi-resolution analysis, DWT separates a signal into low-frequency and high-frequency components across different scales. Supraja et al. [8] demonstrated the effectiveness of wavelet thresholding in enhancing ECG signal quality. Yadav et al. [9] advanced this technique by applying non-local filtering in the wavelet transform domain, which enhances the preservation of critical ECG features during noise removal. While these methods can improve signal quality to some extent, they often rely on predefined assumptions about noise characteristics and may struggle with complex or overlapping noise sources.

Recently, deep learning approaches have shown great promise in addressing these challenges due to their ability to learn intricate patterns in data without the need for manual feature extraction. In 2008, Vincent et al. [10] proposed an architecture based on autoencoder (AE), improved into a denoising autoencoder (DAE), which demonstrated better generalization ability than traditional digital signal processing denoising algorithms. Building on this, Peng Xiong et al. [11] developed a DAE architecture based on Deep Neural Networks (DNNs), utilizing four fully connected layers and sigmoid activation functions in both the encoder and decoder. This architecture offered the advantage of simplicity, but its performance in high-noise scenarios was limited. Chiang et al. [12] introduced a Fully Convolutional DAE (FCN-DAE), which replaced fully connected layers with convolutional layers, significantly reducing the parameter count and computational cost while maintaining decent denoising capabilities. Despite these advancements, the denoising performance of these architectures remained insufficient in scenarios with strong noise.

To address temporal dependencies in sequential data like ECG signals, Hochreiter et al. [13] proposed the Long Short-Term Memory (LSTM) neural network. Building on this, Dasan et al. [14] combined LSTM with DAE to propose the CNN-LSTM DAE, embedding an LSTM module in the encoder’s final layer. This hybrid architecture was able to learn temporal correlations within ECG signals, enabling higher-quality signal reconstruction. However, introducing the LSTM module increased the risk of overfitting in high-noise environments, which negatively impacted denoising performance. Jhang et al. [15,16] further presented innovations with two architectures: the Low-Memory Shortcut Connection DAE (LMSC-DAE) [15] and the Channel-wise Average Pooling with Pixel-Shuffle DAE (CPDAE) [16]. Both architectures incorporated shortcut connections in the encoder and decoder, effectively reconstructing high-quality ECG signals and achieving superior denoising performance. Among existing approaches, CPDAE demonstrated the best overall results, but challenges persist in scenarios with significant noise interference.

Additionally, generative adversarial networks (GANs) have also been explored for ECG denoising tasks. GAN-based methods employ a generator-discriminator framework, where the generator attempts to produce clean ECG signals from noisy inputs, and the discriminator evaluates the quality of the reconstructed signals. This adversarial training process encourages the generator to learn a robust mapping for noise removal. Seo et al. [17] proposed an ECG-GAN framework that successfully denoised ECG signals while preserving critical waveform features. This method achieved superior performance compared to traditional denoising autoencoders and wavelet-based techniques. Wang et al. [18] proposed an improved conditional GAN (CGAN)-based framework for ECG denoising, which features a generator composed of an optimized convolutional autoencoder and a discriminator with four convolutional layers and one fully connected layer. The results achieved an average signal-to-noise ratio (SNR) exceeding 39 dB for both single and mixed noise scenarios, and improved the classification accuracy of four cardiac diseases by over 32% under severe noise conditions.

Recently, inspired by the transformer architecture initially proposed by Vaswani et al. [19] for natural language processing, Meng Chen et al. [20] introduced the transformer-based convolutional denoising autoencoder (TCDAE). This architecture combined a transformer encoder with a convolutional DAE, placing the transformer module in the encoder’s final layer to better capture temporal correlations between features. Compared to CNN-LSTM DAE, TCDAE more effectively incorporated temporal dependencies, which achieves enhanced denoising and reconstruction performance for ECG signals. However, CPDAE still holds an edge in extreme noise scenarios, which indicates that there is still room for improvement in transformer-based approaches.

In summary, advancements in ECG denoising have evolved from traditional filtering techniques to deep learning-based methods, each with unique strengths and limitations. While EMD and DWT improve signal decomposition, they struggle with mode mixing and rely on predefined assumptions about noise characteristics. Deep learning approaches, such as DAEs, have shown promise but often face challenges in generalization under high-noise conditions. GAN-based frameworks improve noise removal and feature preservation but suffer from training instability and high data requirements. Transformer-based architectures effectively capture temporal dependencies but are computationally intensive and less robust in extreme noise scenarios. To address these limitations, this paper introduces a feature-shuffle multi-head attention autoencoder (FMHA-AE), which leverages both feature-shuffling and multi-head self-attention to efficiently remove noise, particularly EM noise, from ECG signals. The proposed model improves signal reconstruction quality and provides a more accurate tool for clinical diagnosis, thus lowering medical costs and improving treatment outcomes. The principal contributions of this work can be summarized as follows:

1. This study introduces the feature-shuffle multi-head attention autoencoder (FMHA-AE) architecture, a cutting-edge framework designed to address the challenges of ECG signal denoising. It outperforms transformer-based architectures by achieving superior denoising performance, particularly in improving signal-to-noise ratio (SNR_imp_) and reducing percentage root mean square difference (PRD), which makes it highly effective for removing challenging noise types such as EM noise;
2. We propose a novel FMHA-AE architecture that incorporates three key components: the feature-shuffle multi-head self-attention (FMHSA) encoder, the multi-head self-attention (MHSA) shortcut, and the feature-shuffle multi-head cross-attention (FMHCA) decoder. This model effectively removes noise, while preserving critical ECG features. By leveraging multi-head attention and feature-shuffling mechanisms, the architecture ensures robust feature extraction and reconstruction, significantly enhancing signal quality for clinical diagnostics;
3. We evaluate the proposed FMHA-AE on MIT-BIH Arrhythmia Database (MITDB) [21] as the source of clean ECG signals and the MIT-BIH Noise Stress Test Database (NSTDB) [22] for noisy signals. The experimental results demonstrate that the model exhibits exceptional denoising and signal reconstruction capabilities under extremely noisy conditions (SNR_in_ from −6 to 24 dB), which achieves an average SNR improvement (SNR_imp_) of 25.34 dB and a percentage root mean square difference (PRD) of 10.29%, which outperforms existing state-of-the-art methods in both signal quality improvement and robustness.

The remainder of this paper is organized as follows. Section 2 reviews related works on feature-shuffle and residual blocks. Section 3 provides a detailed explanation of the proposed FMHA-AE architecture. Section 4 discusses the datasets, training parameters, and experimental results. Finally, Section 5 summarizes the findings and concludes the paper.

## 2. Related Works

### 2.1. Residual Block

**Figure 1:**
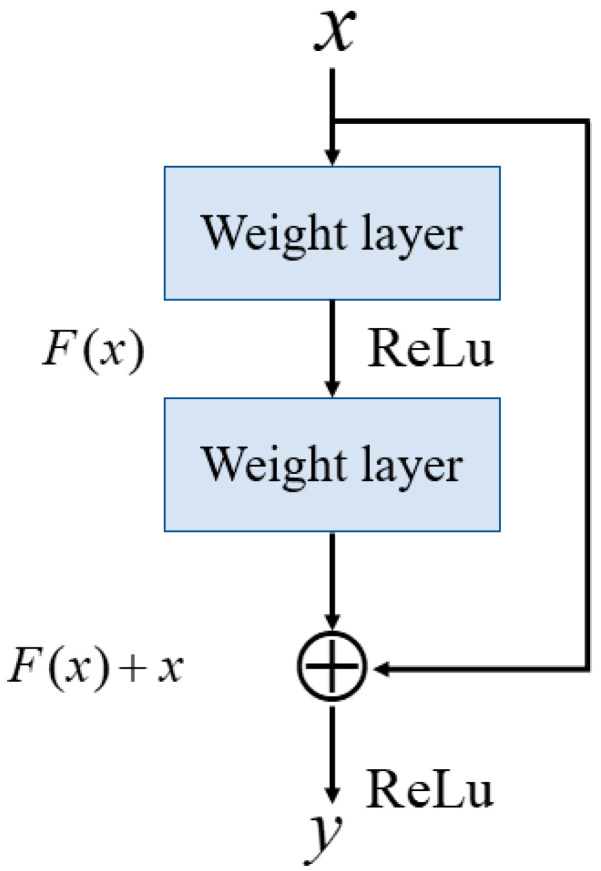
Core framework of residual block [23].

Deep neural networks are prone to issues such as vanishing gradients, which can hinder training efficiency and degrade model performance, especially as the network depth increases. To address this challenge, residual blocks were introduced by He et al. in the ResNet architecture [23], which enables the effective training of very deep networks by incorporating skip connections. A residual block introduces a direct connection (shortcut) that bypasses one or more layers, which allows the input to be directly added to the output of these layers as shown in Figure 1. It can be represented by Equation (1),

(1) y=ReLU(F(x)+x),

where x is the input to the residual block. F(x) represents the transformation function (e.g., convolution) applied to the input. ReLU is the activation function applied to the summed output.

### 2.2. Feature Shuffle

**Figure 2:**
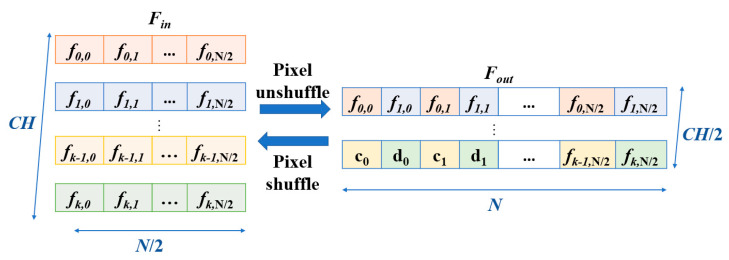
Feature unshuffle and feature shuffle illustration.

Jheng et al. [16] introduced the use of channel-wise average pooling and 1D pixel-shuffle denoising autoencoder for effectively removing electrode motion artifacts in ECG signals. However, the pixel-shuffle technique, commonly used in image processing [24], could be adapted to 1D biomedical data for precise denoising while preserving critical features. Inspired by their work, this paper extends and refines the application of these techniques in ECG denoising tasks. In this study, we employ 1D feature unshuffle/shuffle to rearrange features. Nevertheless, we recognize that the term “pixel” in pixel unshuffle/shuffle may not accurately reflect its functionality when applied to 1D data. The term “pixel” is inherently associated with image-related tasks, making its usage in 1D data somewhat misleading. We suggest the term feature sample, which better captures its purpose and application in 1D data processing. Consequently, this paper adopts the terminology feature unshuffle/shuffle to describe its use in 1D contexts. The illustration of the feature is as shown in Figure 2. Given $F_{in}$ consisting of *CH* input channels A,B,…,C,D, each of size is $1\times \frac{N}{2}$, the transformation rearranges these channels into output channels, each of size 1×N. The interleaving operation alternates elements from consecutive pairs of input channels, which creates the output $F_{out}$. The formulation of feature unshuffle can be represented by Equation (2).

(2) $$F_{out}[k,i]=\left\{\begin{array}{l} F_{in}[2k,i/2],\;\mathrm{if}\;i\;\mathrm{is}\;\mathrm{even},\\ F_{in}[2k+1,i/2],\;\mathrm{if}\;i\;\mathrm{is}\;\mathrm{odd}. \end{array}\right.$$

where i∈[0,N−1] and $k\in [0,\frac{CH}{2}-1]$. The transformation of feature shuffle can be represented by Equation (3).

(3) $$\begin{array}{c} F_{in}[2k,j]=F_{out}[k,2j]\\ F_{in}[2k+1,j]=F_{out}[k,2j+1] \end{array},\;\mathrm{for}\;j\in [0,\frac{N}{2}-1].$$

Many denoising neural network architectures employ pooling or stride-based operations to reduce feature length for downsampling [25,26]. In contrast, this study utilizes 1D feature unshuffle to rearrange features, which converts spatial dimension information into higher-dimensional channel representations. In the proposed method, when paired with point-wise convolution, it facilitates a downsampling effect by halving the feature length while keeping the channel dimensions intact. This approach not only simplifies feature transformations but also preserves vital information for improved signal reconstruction and denoising performance.

## 3. Methods

The feature-shuffling multi-head attention autoencoder (FMHA-AE) is designed to enhance the process of ECG signal denoising by combining feature-shuffling and multi-head self-attention mechanisms. The architecture consists of an encoder-decoder structure where the core components of the model operate in three stages: the feature-shuffle multi-head self-attention (FMHSA) encoder, multi-head self-attention (MHSA) shortcut, and feature multi-head cross-attention (FMHCA) decoder. The model incorporates three distinctive features that contribute to its effectiveness: (1) the integration of residual blocks and feature-unshuffle in the FMHSA encoder to improve feature extraction and noise suppression, (2) the use of MHSA shortcuts to transmit critical feature information across stages, enhancing reconstruction accuracy, and (3) the employment of FMHCA in the decoder to refine the reconstruction process by leveraging cross-attention mechanisms. These features collectively enable the model to address the challenges of noisy ECG signals, as detailed in the subsequent explanation.

Noisy ECG signals are first sent to the input layer to expand the channels. The FMHSA Encoder extracts high-dimensional clean ECG features, which are then reconstructed in the FMHCA Decoder. Additionally, information from the FMHSA Encoder is transmitted to the FMHCA Decoder through the MHSA Shortcut to supplement the progressively reduced feature content, thereby enhancing the reconstruction quality. The FMHSA encoder integrates residual blocks, feature unshuffle, point-wise convolution, and MHSA to enhance noisy ECG signal feature extraction. The residual block mitigates vanishing gradient issues, while feature unshuffle transforms spatial dimensions into higher-dimensional channel representations for effective noise suppression. The MHSA block leverages multi-head mechanisms to capture temporal and spatial dependencies across features, improving robustness in denoising.

**Figure 3:**
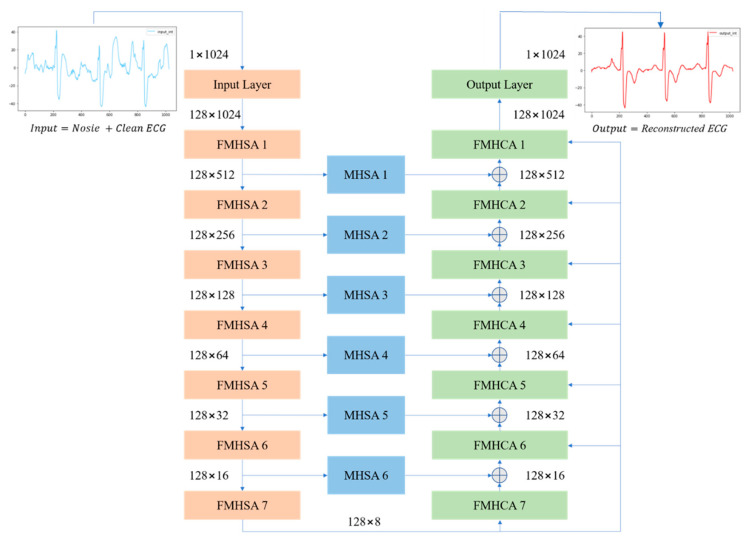
Overall architecture diagram of FMHA-AE.

Figure 3 presents an overview of the proposed FMHA-AE model, which contains seven encoders, seven decoders, and six shortcut layers. The noisy ECG signals input size is 1 × 1024, and the feature channels has been expended to 128 through the convolutional layer. The encoder’s output channel size remains at 128, and the feature size has decreased by 50% each layer. After the 7th-layer encoder operation, the feature size is reduced from 1024 to 8. Conversely, the decoder operation process is designed to progressively upsample the feature size, which is double for each layer. After the 7th-layer decoder operation, the feature size is fully restored to its original dimension of 1024. The final output size of the decoder is 128 × 1024, which results in the reconstructed ECG signal with the same spatial resolution as the input. To further enhance the noise reduction performance of the proposed FMHA-AE, a multi-head attention operation is inserted between the encoder and decoder to transfer more feature information which is helpful for the decoder to reconstruct the noise-less ECG signals. The architecture’s design ensures robust feature extraction and reconstruction across multiple stages, as detailed in Figure 3.

Table 1 summarizes the architecture of the proposed model, which includes the number of trainable parameters, input size, and output size for each layer. The encoder consists of an input layer followed by seven FMHSA encoder blocks, which progressively reduces the temporal dimension by a factor of 2 while maintaining the feature channel dimension at 128. Each FMHSA encoder block has 395,264 trainable parameters. The shortcut connections, which is implemented as MHSA blocks, preserve intermediate representations for multiscale reconstruction and have 198,272 trainable parameters for each block. The decoder, which consists of seven FMHCA decoder blocks, reconstructs the feature dimensions back to the original temporal size, with each block containing 395,392 parameters. The final output layer maps the reconstructed feature space back to the original single-channel signal, with 164,225 parameters. Overall, the proposed model effectively encodes and reconstructs the 1D ECG signal while leveraging hierarchical feature representations and shortcut connections for improved performance.

### 3.1. Feature-Shuffle Multi-Head Self-Attention (FMHSA) Encoder Architecture Design

**Figure 4:**
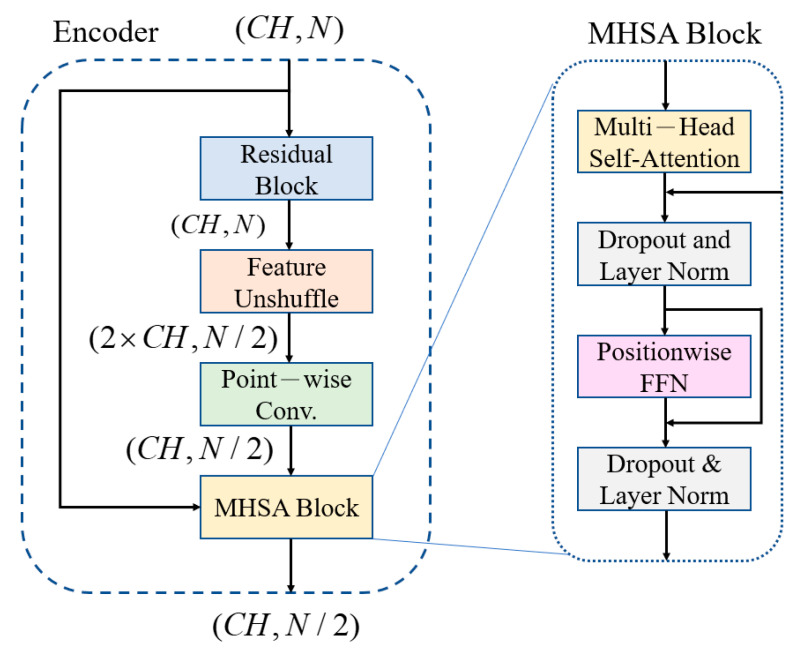
FMHSA encoder and MHSA block architecture diagram.

The noisy inputs are fed into an encoder comprising of a convolutional encoder and seven FMHSA encoders, as shown in Figure 2. First, the input 1 × 1024 noisy ECG signal is fed into the input layer, where a 1 × 1 1D convolution (conv.) is used to expand the channels to 128. After passing through seven FMHSA encoders, 128 × 8 features are obtained. The FMHSA encoder architecture is shown in Figure 4, which includes the residual block, feature-unshuffle block, point-wise conv. block, and multi-head self-attention (MHSA) block. The residual block plays a critical role in the FMHSA encoder by addressing vanishing gradient issues and enhancing the network’s ability to train effectively for deeper layers. It introduces a shortcut connection that bypasses one or more layers, which allows the input to be directly added to the output of these layers. This design preserves essential input features while enabling deeper representations of the data. The FMHSA encoder begins with a residual block that employs a 1D convolution with a kernel size of 5 and padding of 2 to maintain *CH* channels and feature dimension *N*. The input features are duplicated and processed through two sequential 1D convolutional layers, each followed by an activation function, such as ReLU, to introduce non-linearity. The outputs of these layers are summed with the original input to produce the final output of the residual block. This structure mitigates the risk of vanishing gradients in deep neural networks by maintaining feature stability across layers. Subsequently, a feature-unshuffle layer rearranges the features, which converts the spatial dimension information of the input into a higher channel dimension. Next, a point-wise convolution layer with a kernel size of 1 is applied, which reshapes the data to (2×CH, N/2). This operation facilitates the mixing of information across channels without modifying the spatial dimensions. The combination of this technique with the point-wise convolution effectively reduces the feature length by half while maintaining the channel length unchanged.

**Figure 5:**
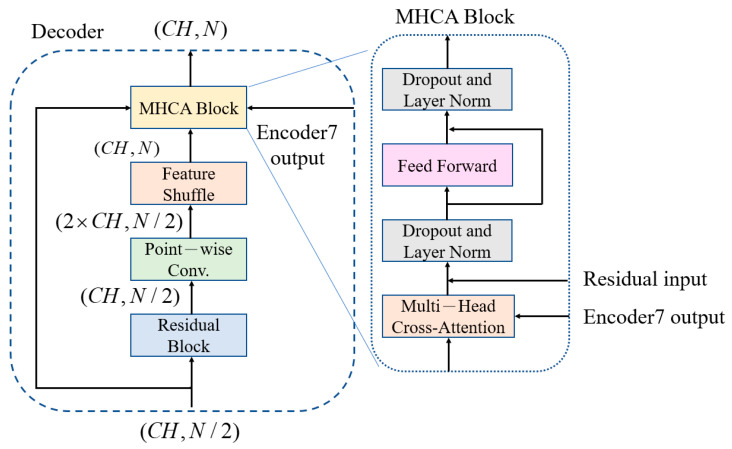
FMHCA decoder block architecture diagram.

Subsequently, the features are input into the MHSA block. MHSA is a core component that can integrate relative positions in different spatial dimensions, as shown in Figure 5. The feature signal X is used as query (Q), key (K), and value (V). They pass through three different linear layers $W^{Q}$, $W^{K}$, $W^{V}$, where $X\in {\mathbb{R}}^{N\times CH}$, $W^{Q}$, $W^{K}$, $W^{V}$$\in {\mathbb{R}}^{CH\times d\_k}$, to obtain the weight matrices. The feature channels are evenly divided into multiple heads, each with its $Q_{i}$, $K_{i}$, $V_{i}$ matrices. This allows the feature to focus on different aspects of information in different spatial dimensions. Using a single attention function with CH dimensional queries, keys, and values can be improved by linearly projecting the queries, keys, and values h times through different learned linear projections to d_k, d_q, d_v. Equations (4) and (5) show this process, where $Q_{i}$, $K_{i}$, $V_{i}$$\in {\mathbb{R}}^{N\times d\_k}$ and h represents of head number.

(4) $$d\_k=\frac{CH}{h}$$

(5) $$Q_{i}=QW_{i}^{Q},\;K_{i}=KW_{i}^{K},\;V_{i}=VW_{i}^{V}$$

**Figure 6:**
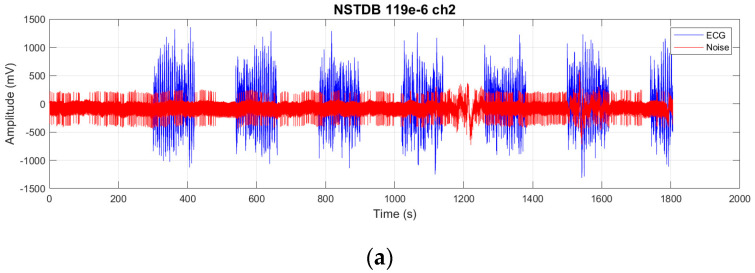

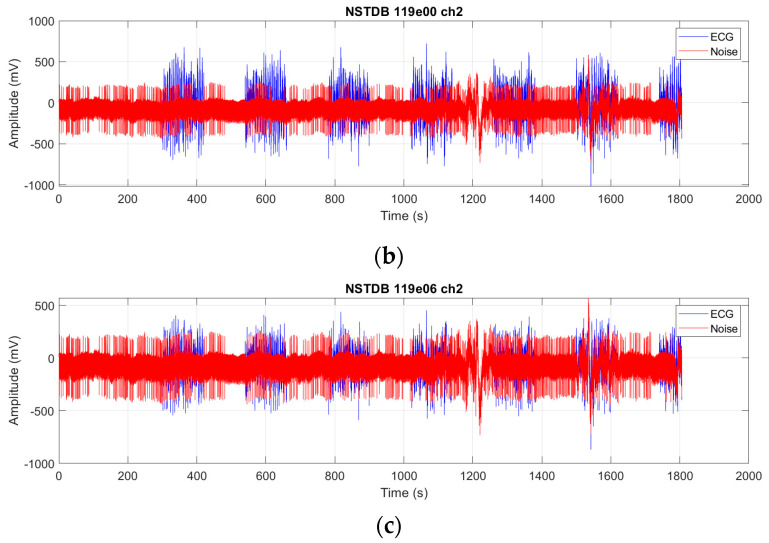
Record 119 with different levels of noise: (**a**) clean signal with −6 dB noise, (**b**) clean signal with 0 dB noise, (**c**) clean signal with 6 dB noise.

**Figure 7:**
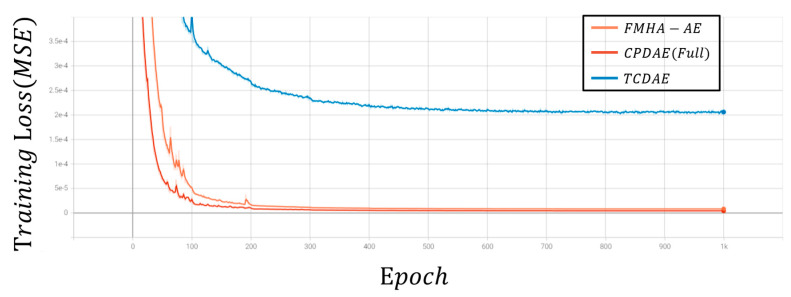
Loss curve during the training phase.

Each head’s relative position weight output matrix is calculated by scaling the dot product of $Q_{i}$ and $k_{i}^{T}$ with $1/\sqrt{d\_k}$, then applying the softmax function to get the attention weight matrix, and finally multiplying with $V_{i}$ to get the final output matrix, as shown in Equation (6), where $head_{i}$$\in {\mathbb{R}}^{N\times d\_k}$. The outputs of all heads are concatenated together and projected back to the original dimension using a linear layer, as shown in Equation (7), where $W^{O}$$\in {\mathbb{R}}^{CH\times d\_k}$. The final result is $MultiHead(Q,K,V)\in {\mathbb{R}}^{N\times CH}$. The overall process is illustrated in Figure 6 and Figure 7.

(6) $$head_{i}=softmax(\frac{Q_{i}K_{i}^{T}}{\sqrt{d_{k}}})V_{i}$$

(7) $$MultiHead(Q,K,V)=concat(head_{1},\ldots ,head_{h})W^{O}$$

Since the operations performed by MHSA are linear transformations, its ability to learn complex feature relationships is not as strong as that of nonlinear transformations. Therefore, after the MHSA, we input the features into a feed-forward network (FFN). The formula is shown in Equation (8). *W*_1_ and *W*_2_ are weight matrices of the two fully connected layers, and *b*_1_ and *b*_2_ are respective biases. The operation $\max(0,XW_{1}+b_{1})$ applies a ReLU activation function. By employing these nonlinear activation functions, we can significantly enhance the model’s expressive power, which enables it to capture and learn more complex and detailed feature relationships. This step not only enriches the feature representations but also improves the overall network’s denoising and reconstruction performance.

(8) $$FFN(X)=\max(0,XW_{1}+b_{1})W_{2}+b_{2}$$

### 3.2. Multi-Head Self-Attention (MHSA) Shortcut Architecture

As the encoder extracts features layer by layer, some subtle but indispensable features may vanish with the deepening of the network layers. This can result in the reconstructed signal not fully approximating the clean signal [27]. To address this issue, an MHSA shortcut is added between the encoder and the decoder, which allows the decoder to receive more information from the encoder during signal reconstruction, thereby enhances the reconstruction quality. The FMHSA encoder in this paper focuses on learning correlations between different time steps. The MHSA shortcut also utilizes the MHSA block, but unlike the FMHSA encoder, the MHSA shortcut aims to learn correlations between different channels. This approach allows the decoder to not only leverage the features across time steps but also more effectively integrate crucial information from different channels, thereby further improving the performance in denoising and reconstruction. The MHSA architecture is illustrated in Figure 4.

### 3.3. Feature-Shuffle Multi-Head Cross-Attention (FMHCA) Decoder Architecture Design

The architecture of the FMHCA decoder is shown in Figure 5. The input signal first undergoes preliminary extraction through a residual block. Next, the signal passes through a point-wise conv., which doubles the number of channels to increase the feature representation capacity. Following this, the signal enters the feature-shuffle block, which converts high-dimensional channel features into low-dimensional spatial features, as illustrated in Figure 2. These two modules enable the sequence length to double while keeping the number of channels unchanged, which achieves an upsampling effect. This series of operations not only assists in the layer-by-layer reconstruction of the signal but also effectively enhances the quality of the reconstructed signal, which makes it closer to the original clean ECG signal.

Next, the features are input into the multi-head cross-attention (MHCA) block, as illustrated in Figure 5. The MHCA module has two inputs: one is the output from the feature shuffle, denoted as $F\in {\mathbb{R}}^{F\_len\times CH}$, and the other is the output from the seventh layer of the MHSA encoder, denoted as $E\in {\mathbb{R}}^{E\_len\times CH}$. F passes through the linear multi-head weight matrix $W_{i}^{Q}$ to form multiple heads of $Q_{i}$, while E passes through the linear multi-head weight matrices $W_{i}^{K}$, $W_{i}^{V}$ to obtain $K_{i}$, $V_{i}$, as shown in Equation (9). Next, $Q_{i}$, $K_{i}$, $V_{i}$ are used to compute the attention weight matrix as shown in Equation (10). Finally, all head output matrices are concatenated and projected back to the original dimension using the linear weight matrix $W^{O}$, as shown in Equation (11). The significance of this lies in that the output of the final layer of the decoder includes the high-dimensional features of the entire ECG signal. These features help the decoder better understand the comprehensive characteristics and structure of the signal. When these high-dimensional features are input into each layer of the MHCA module of the decoder, the decoder can reference these comprehensive features during each step of signal reconstruction, which results in a more accurate signal restoration.

(9) $$Q_{i}=FW_{i}^{Q},K_{i}=EW_{i}^{K},V_{i}=EW_{i}^{V}$$

(10) $$head_{i}=softmax(\frac{Q_{i}K_{i}^{T}}{\sqrt{d_{k}}})V_{i}$$

(11) $$MultiHead(Q,K,V)=concat(head_{1},\ldots ,head_{h})W^{O}$$

Similarly, each MHCA block is followed by a feed-forward network to improve the learning capacity of the nonlinear components. After processing through seven layers of MHCA decoders, a 1 × 1 conv. layer is applied to restore the signal back to the original signal dimensions, which ensures that the final reconstructed ECG signal is consistent with the initial signal.

## 4. Experimental Results

### 4.1. Dataset Preparation and Preprocessing for ECG Signal Denoising

This paper uses the publicly available MIT-BIH Arrhythmia Database (MITDB) [21], jointly collected by the Massachusetts Institute of Technology (MIT) and Beth Israel Deaconess Medical Center in Boston, as the source of clean ECG signals. The database includes 48 half-hour dual-channel ambulatory ECG recordings, each channel sampled at 360 samples per second (sample rate 360 Hz), with 11-bit resolution over a 10 mV range. Each record is independently annotated by two or more cardiologists.

The noisy ECG signals are sourced from the MIT-BIH Noise Stress Test Database (NSTDB) [22]. This dataset applies baseline wander (BW), muscle artifacts (MAs), and electrode motion (EM) to records 118 and 119 from MITDB. EM is considered the most challenging noise to remove because it can mimic the waveform of ectopic beats. The noise intensity is available in six different levels (−6 dB, 0 dB, 6 dB, 12 dB, 18 dB, 24 dB). EM noise is added for the first 5 min, followed by alternating 2-min segments of clean and noisy signals. As shown in Figure 6, the orange signal represents the clean MITDB signal, while the blue signal represents the noisy NSTDB signal. It is evident that the noise at −6 dB is stronger, whereas the noise at 24 dB is weaker. This paper utilizes NSTDB signals containing EM noise as input and the clean MITDB signals as ground truth. The error between these two datasets is calculated to adjust model weights, facilitating the training of the denoising architecture. The input signals are segmented into 1024-length segments, with no overlap between segments, resulting in 6888 segments. These segments are split into training and test sets with an 80%:20% ratio. To reduce the influence of DC offset on PRD, all segments had their DC offset removed during preprocessing, and the data were normalized to a range between −1 and 1. The 1024-sample length aligns with the 360 Hz sampling rate from our prior acquisition system [15], capturing 2-4 heartbeat peaks for effective denoising, while DC offset removal and normalization enhance model robustness as validated by the reported SNR and PRD improvements.

### 4.2. Training Environment and Hyperparameter Settings

The training environment for the architecture proposed in this paper is shown in Table 2. Training was conducted using an Nvidia RTX 3090 GPU with the PyTorch 1.6 framework. The training hyperparameters are listed in Table 3. The model was trained using the mean square error (MSE) loss function, with a batch size of 32. The learning rate is managed with a step-LR schedule, which starts at $1\times 10^{-4}$ and halves every 200 epochs. The Adam optimizer was employed for a total of 1000 epochs.

### 4.3. Evaluation Metrics

This paper uses the improvement in signal-to-noise ratio (SNR_imp_) and the percentage root mean square difference (PRD) as evaluation metrics. Here, N represents the total length of the signal, $x_{i}$ represents the clean ECG signal, ${\hat{x}}_{i}$ represents the denoised ECG signal, and ${\tilde{x}}_{i}$ represents the noisy ECG signal. SNR_in_ (as shown in Equation (12)) is calculated between the clean signal and the noisy signal, while SNR_out_ (as shown in Equation (13)) is calculated between the clean signal and the denoised signal. SNR_imp_ (as shown in Equation (14)) represents the denoising effect as the difference between the SNR values before and after denoising. A negative value indicates unsuccessful denoising, while a positive value indicates successful denoising, with larger values reflecting better denoising performance. PRD (as shown in Equation (15)) represents the reconstruction quality between the clean signal and reconstructed signal, where smaller values indicate a closer match to the clean signal. Additionally, the root mean square error (RMSE) is employed as a metric to evaluate the accuracy of the denoised signal relative to the clean signal, calculated as shown in Equation (16).

(12) $${\mathrm{SNR}}_{in}=\frac{\sum_{i=1}^{N}x_{i}^{2}}{\sum_{i=1}^{N}{\left(x_{i}-{\tilde{x}}_{i}\right)}^{2}}$$

(13) $${\mathrm{SNR}}_{out}=\frac{\sum_{i=1}^{N}x_{i}^{2}}{\sum_{i=1}^{N}{\left(x_{i}-{\hat{x}}_{i}\right)}^{2}}$$

(14) $${\mathrm{SNR}}_{imp}=10\;∙{\;\log}_{10}{(\mathrm{SNR}}_{out})-10\;∙{\;\log}_{10}{(\mathrm{SNR}}_{in})$$

(15) $$\mathrm{PRD}=\sqrt{\frac{\sum_{i=1}^{N}{\left(x_{i}-{\hat{x}}_{i}\right)}^{2}}{\sum_{i=1}^{N}{x_{i}}^{2}}}\times 100\%$$

(16) $$\mathrm{RMSE}=\sqrt{\frac{1}{N}\sum_{i=1}^{N}{\left(x_{i}-{\hat{x}}_{i}\right)}^{2}}$$

### 4.4. Implementation Comparison

This paper compares the proposed method with the following seven existing architectures: (1) DNN-DAE [11], (2) CNN-DAE, (3) FCN-DAE [12], (4) CNN-LSTM-DAE [14], (5) LMSC-DAE [15], (6) CPDAE [16], (7) TCDAE [20].

The DNN-DAE model is structured with 10 fully connected layers, where the nodes halve from 512 to 32 over the first five layers and then double back up to 1024 in the remaining layers: 512, 256, 128, 64, 32, 64, 128, 256, and 1024, respectively. The CNN-DAE employs six convolutional layers in the encoder and six transposed convolutional layers plus two fully connected layers in the decoder. Similarly, the FCN-DAE contains six convolutional layers in the encoder and seven transposed convolutional layers in the decoder. The CNN-DAE can thus be seen as a modification of the FCN-DAE with additional fully connected layers to integrate global information. The CNN-LSTM-DAE uses eight convolutional layers and five max-pooling layers to extract features in the encoder and incorporates an LSTM module at the end of the encoder to learn temporal correlations in the sequence data. The decoder includes eight convolutional layers, six upsampling layers, and one fully connected layer to reconstruct the ECG signal. The LMSC-DAE consists of two convolutional layers, eight encoder layers, eight decoder layers, and seven LMSC layers. CPDAE includes two convolutional layers, seven encoder layers, seven decoder layers, and six Channel-wise average pooling (CWAP) layers. The TCDAE architecture combines a convolutional encoder and a Transformer encoder. The convolutional encoder comprises three convolutional layers, each followed by a gated convolution. The Transformer encoder consists of one position embedding layer and six Transformer modules. This model employs a modified weighted Huber Loss as its loss function to enhance performance under challenging conditions.

### 4.5. Experimental Results

**Figure 8:**
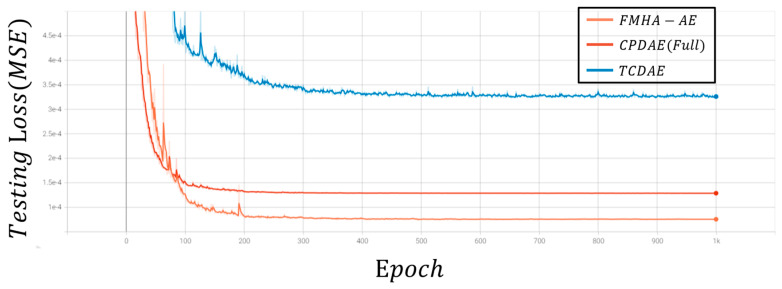
Loss curve during the testing phase.

Figure 7 and Figure 8 depict the average loss curves for CPDAE (Full) [16], TCDAE [20], and FMHA-AE on the training and test sets. During the training phase, the loss decreases consistently over the first 400 epochs before stabilizing, which informed the decision to set the total training epochs to 1000. Notably, FMHA-AE converges at a slower rate during the training phase compared to CPDAE (Full). However, it ultimately achieves a similar level of loss reduction. In the testing phase, FMHA-AE exhibits superior generalization performance, which outperforms CPDAE (Full) and TCDAE in denoising and reconstructing unfamiliar signals with greater accuracy.

#### 4.5.1. Computational Efficiency and Performance

Table 4 compares various model architectures based on their parameters and multiplication-addition calculations (MACs), which brings attention to differences in computational efficiency and resource requirements. The DNN-DAE model has 1.4 million (M) parameters with an equivalent 1.4 M MACs, while the CNN-DAE model reduces parameters to 1.12 M but increases MACs to 13.27 M. The FCN-DAE, with only 78.44 K parameters, requires a higher 25.08 M MACs, showing efficiency in parameter use but greater computational demand. In contrast, the CNN-LSTM-DAE model, combining convolutional and recurrent layers, has 10.92 M parameters and 46.69 M MACs. The LMSC-DAE is the most lightweight with 63.62 K parameters and 12.36 M MACs. The CPDAE (Full) model, with 2.69 M parameters and a substantial 355.01 M MACs, reflects high resource intensity. The TCDAE model, with 0.31 M parameters and 60 M MACs, focuses on computational demand rather than parameter count, while the FMHA-AE model, exhibiting the highest computational complexity, has 4.28 M parameters and 1640 M MACs, indicative of its multi-head attention operations. While FMHA-AE demonstrates superior performance across all evaluation metrics, it is noteworthy that the model’s higher parameter count (4.28 M) and computational complexity (1640 M MACs) may pose challenges for deployment in resource-constrained environments. However, these trade-offs are justified by its exceptional denoising capabilities, particularly under low-SNR conditions (−6 dB). The results suggest that FMHA-AE’s advanced mechanisms, which includes feature-shuffling and multi-head self-attention, effectively enhance its robustness and accuracy, making it a reliable tool for clinical diagnostics where computational resources are available.

Table 4 further demonstrates the balance between computational efficiency and performance. For example, lighter models such as LMSC-DAE (63.62 K parameters and 12.36 M MACs) achieve moderate PRD values but lack the robustness observed in FMHA-AE under severe noise conditions. In contrast, FMHA-AE’s architecture is specifically designed to handle overlapping and complex noise patterns, as evidenced by its average PRD of 11.32%, compared to LMSC-DAE’s 47.02%.

#### 4.5.2. Measurement of Channel Expansion on Denoising Performance

To address the role of the channel expansion operation in the input convolutional layer, which increases the feature channels to 128 (resulting in an output size of 128 × 1024 before entering the encoder), we investigated its impact on noise reduction quality through an ablation study. Specifically, the number of channels in this layer was varied to 64, 128 (the original configuration), and 256, while all other architectural parameters were kept constant, including seven encoder and seven decoder layers, four multi-head attention heads, and proportionally scaled feedforward dimensions (d_ff = 4 × d_model). Here, d_ff denotes the hidden dimension of the feedforward network in each attention block, which is typically set to four times the model dimension to enhance the model’s representation capacity (d_model). The models were trained and evaluated on the same MITDB and NSTDB across input SNR levels from −6 dB to 24 dB.

The results demonstrate that increasing the number of channels enhances the model’s capacity to capture complex features, thereby improving denoising performance as measured by SNR_imp_ and PRD. However, this comes at the expense of higher computational complexity, including more trainable parameters and longer training times. The 128-channel configuration provides an optimal balance, achieving a PRD of 10.29%, SNR_imp_ of 25.34 dB, and RMSE of 0.0037, outperforming the 64-channel model (PRD: 11.52%, SNR_imp_: 25.22 dB, RMSE: 0.00419) in high-noise scenarios while avoiding the excessive resource demands of the 256-channel variant (PRD: 10.76%, SNR_imp_: 25.49 dB, RMSE: 0.00403), confirming its efficiency in noise reduction. Detailed comparisons are presented in Table 5. The total training times for the different channel configurations are as follows: 20,188.35 s for 64 channels, 32,375.13 s for 128 channels, and 68,500.74 s for 256 channels, reflecting the increased computational demand with higher channel numbers.

#### 4.5.3. Measurement of Head Numbers

To determine the optimal number of heads for FMHA-AE, experiments were conducted using different configurations of head numbers (2, 4, 8, and 16). The evaluation metrics PRD, RMSE, and SNR_imp_ were calculated based on the mean values of the test data, as detailed in Table 6. The results indicate that the PRD gradually improves as the head number increases, which reaches its lowest value of 10.29% at four heads, before slightly increasing at higher head numbers. Similarly, RMSE decreases from 0.00412 at two heads to a minimum of 0.0037 at four heads, followed by a slight upward trend with additional heads. In contrast, SNR_imp_ remains relatively stable across all configurations, peaking at 25.34 dB with 4 heads and showing minimal variation at 8 and 16 heads.

#### 4.5.4. Performance Evaluation Compared to Non-DAEs

Conventional signal processing techniques, including Finite Impulse Response (FIR) filters, Infinite Impulse Response (IIR) filters, and Discrete Wavelet Transform (DWT), are extensively used for signal denoising due to their straightforward implementation and clear interpretability. The FIR filter was designed with a Hanning window of order 101, incorporating cutoff frequencies of 0.67 Hz (high-pass) and 40 Hz (low-pass) to effectively mitigate baseline wander and high-frequency noise. The IIR filter adopted a fourth-order Butterworth configuration with identical cutoff frequencies, striking a balance between computational efficiency and denoising effectiveness. The DWT approach utilized a Daubechies 6 (db6) wavelet with a nine-level decomposition, where the highest, second-highest, and lowest frequency coefficients were nullified to eliminate noise while maintaining the integrity of ECG signal morphology.

The performance of these methods, alongside the proposed FMHA-AE, was evaluated across varying input noise levels, as shown in Table 7. The SNR_imp_ metric quantifies the improvement in signal-to-noise ratio post-denoising, with higher values indicating better noise suppression. The proposed FMHA-AE consistently outperforms traditional methods across all input SNR levels, achieving an average SNR_imp_ of 25.34 dB, compared to 1.73 dB for FIR, 1.83 dB for IIR, and 2.18 dB for DWT. Notably, traditional methods exhibit diminishing performance at higher input SNR levels (e.g., 24 dB), where they occasionally yield negative SNR_imp_ values, indicating signal degradation. In contrast, FMHA-AE maintains robust performance, even at high input SNR levels. As shown in Table 8, the PRD metric quantifies the reconstruction error between the clean and denoised ECG signals, with lower values indicating better preservation of signal quality. The proposed FMHA-AE significantly outperforms traditional methods across all input SNR levels, achieving an average PRD of 10.29%, compared to 178.8% for FIR, 175.9% for IIR, and 174.5% for DWT. Notably, traditional methods exhibit substantially higher PRD values, particularly at lower input SNR levels (e.g., −6 dB), reflecting poor noise suppression and signal distortion. In contrast, FMHA-AE maintains robust performance with a low PRD even at challenging noise levels.

#### 4.5.5. Performance Evaluation Compared to Baseline Architecture

The model uses 4-head FMSHA-AE in each encoder and decoder. Table 9 compares the SNR improvement (SNR_imp_) in decibels (dB) achieved by the proposed architecture (FMHA-AE) and other baseline architectures across a range of input SNR levels (−6 dB, 0 dB, 6 dB, 12 dB, 18 dB, and 24 dB), along with the overall improvement. FMHA-AE demonstrates superior performance, which consistently achieves the highest SNR_imp_ values across all input levels. The average SNR_imp_ for FMHA-AE is 25.34 dB, which significantly outperforms the other architectures, such as CPDAE (Full) at 20.48 dB and LMSC-DAE at 10.37 dB, which depicts the effectiveness in improving signal quality.

Similarly, Table 10 presents a comparison of the PRD for the proposed architecture and baseline methods over the same range of input SNR levels. The FMHA-AE again shows the best performance, which achieves the lowest PRD values across all input SNR levels, with an average PRD of 10.29%. This result is significantly better than the next best architecture, CPDAE (Full), which has an average PRD of 18.12%. The higher PRD values of the other models, such as LMSC-DAE (47.02%) and CNN-DAE (64.17%), show that they struggle to minimize reconstruction errors under severe conditions. These results clearly demonstrate the robustness and precision of FMHA-AE in denoising ECG signals, which establish it as a reliable tool for clinical applications where accurate signal reconstruction is crucial.

**Figure 9:**
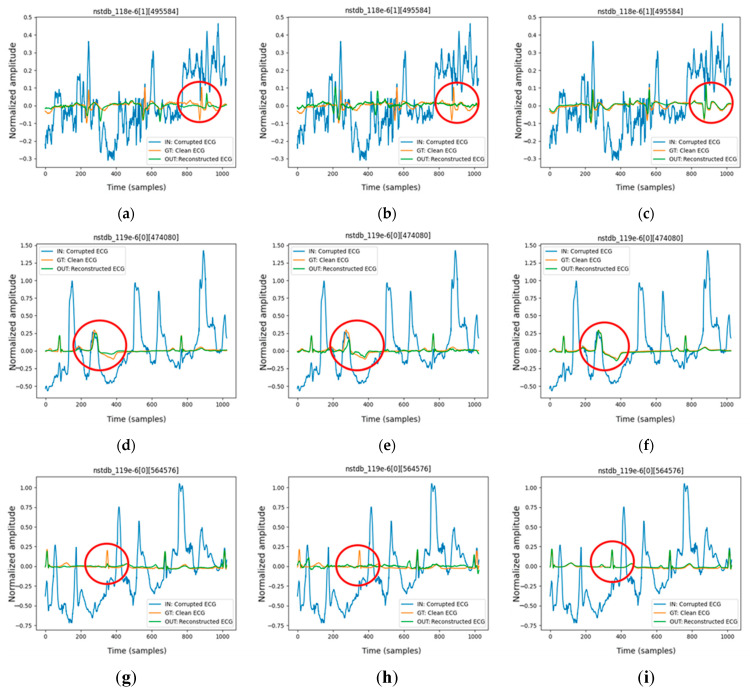
Denoised waveform results at SNR_in_ (−6 dB) for different segments: (**a**) CPDAE (Full), (**b**) TCDAE, and (**c**) FMHA-AE for one segment of NSTDB 118 in lead VI; (**d**) CPDAE (Full), (**e**) TCDAE, and (**f**) FMHA-AE for segment 1 of NSTDB 119 in lead MLII; (**g**) CPDAE (Full), (**h**) TCDAE, and (**i**) FMHA-AE for segment 2 of NSTDB 119 in lead MLII.

Figure 9 presents the denoised ECG waveform results at an input SNR of −6 dB for three architectures: CPDAE (Full), TCDAE, and FMHA-AE. The evaluation includes two leads from the NSTDB database: lead VI in record 118 and lead MLII in record 119. In Figure 9a–c, FMHA-AE demonstrates the closest alignment with the clean ECG signal, effectively reconstructing critical waveform features, particularly in the red-circled regions where CPDAE (Full) and TCDAE exhibit notable distortions. The red circles in Figure 9 delineate specific segments of the denoised ECG waveforms where reconstruction challenges are evident, highlighting areas of poor performance by CPDAE (Full) and TCDAE, characterized by distortions and loss of critical waveform features due to electrode motion noise at −6 dB SNR. In contrast, FMHA-AE effectively mitigates these issues, restoring the signal with high fidelity, as evidenced by its closer alignment with the clean ECG. In Figure 9d–f, FMHA-AE consistently outperforms CPDAE (Full) and TCDAE by preserving key signal characteristics and mitigating noise interference, resulting in a smoother and more accurate reconstruction. Similarly, in Figure 9g–i, FMHA-AE further highlights its robustness under challenging noise conditions, maintaining waveform integrity and minimizing deviations from the clean ECG compared to the other methods. The red-circled regions across the panels emphasize the superior denoising performance of FMHA-AE, which consistently provides the most accurate reconstructions while effectively preserving the morphology of the original ECG signals. Overall, FMHA-AE consistently achieves the highest reconstruction accuracy across all segments and leads, which demonstrates its robustness in handling noisy ECG signals under challenging SNR conditions.

## 5. Conclusions

This study introduces the feature-shuffle and FMHA-AE architecture as a novel and effective solution for ECG signal denoising. By integrating feature-shuffling and multi-head self-attention mechanisms, FMHA-AE significantly enhances signal reconstruction quality, which achieves superior noise reduction metrics compared to existing methods. Experimental results validated on benchmark datasets, which includes MITDB and NSTDB, confirm that the proposed model effectively preserves signal integrity, even under high-noise conditions. Specifically, FMHA-AE achieves an average SNR_imp_ of 25.34 dB and a PRD of 10.29%, which demonstrates its reliability and potential for clinical applications, such as improving diagnostic accuracy and reducing healthcare costs. Future research may focus on optimizing the FMHA-AE architecture for real-time ECG monitoring systems and extending its adaptability to other biosignals, such as electromyography (EMG) or phonocardiogram (PCG). These efforts could further enhance its scalability and applicability in diverse medical scenarios. In conclusion, the FMHA-AE model represents a significant advancement in leveraging deep learning techniques to address critical challenges in medical signal processing, providing a foundation for more accurate and robust biomedical applications.

## Figures and Tables

**Table 1:** Detailed architecture information of FMHA-AE.

Block	Layer	No. of Trainable Parameters	Input Size	Output Size CH × N
—	Input	—	—	1 × 1024
Encoder	Input Layer	164,352	1 × 1024	128 × 1024
	FMHSA Encoder 1	395,264	128 × 1024	128 × 512
	FMHSA Encoder 2	395,264	128 × 512	128 × 256
	FMHSA Encoder 3	395,264	128 × 256	128 × 128
	FMHSA Encoder 4	395,264	128 × 128	128 × 64
	FMHSA Encoder 5	395,264	128 × 64	128 × 32
	FMHSA Encoder 6	395,264	128 × 32	128 × 16
	FMHSA Encoder 7	395,264	128 × 16	128 × 8
Shortcut	MHSA Shortcut 1	198,272	128 × 512	128 × 512
	MHSA Shortcut 2	198,272	128 × 256	128 × 256
	MHSA Shortcut 3	198,272	128 × 128	128 × 128
	MHSA Shortcut 4	198,272	128 × 64	128 × 64
	MHSA Shortcut 5	198,272	128 × 32	128 × 32
	MHSA Shortcut 6	198,272	128 × 16	128 × 16
Decoder	FMHCA Decoder 7	395,392	128 × 8	128 × 16
	FMHCA Decoder 6	395,392	128 × 16	128 × 32
	FMHCA Decoder 5	395,392	128 × 32	128 × 64
	FMHCA Decoder 4	395,392	128 × 64	128 × 128
	FMHCA Decoder 3	395,392	128 × 128	128 × 256
	FMHCA Decoder 2	395,392	128 × 256	128 × 512
	FMHCA Decoder 1	395,392	128 × 512	128 × 1024
	Output Layer	164,225	128 × 1024	1 × 1024
(Reconstructed ECG)	Output	—	—	1 × 1024

**Table 2:** Training environment for the architecture.

Item	Information
CPU	AMD Ryzen 5 5600X
RAM	32 GB
GPU	RTX 3090
Framework	PyTorch 1.6

**Table 3:** Training hyperparameters.

Hyperparameters	
Loss Function	Mean-Square Error (MSE) $MSE=\frac{1}{n}\sum_{i=1}^{n}{\left(x_{i}-{\tilde{x}}_{i}\right)}^{2}$
Batch Size	32
Learning Rate	$1\times 10^{-4}$ (Step-LR)
Optimizer	Adam
Epochs	1000
Heads	4
Hidden size	512

**Table 4:** Comparison of parameters and MACs for various architectures.

Models	Parameters	MACs
DNN-DAE [11]	1.4 M	1.4 M
CNN-DAE	1.12 M	13.27 M
FCN-DAE [12]	78.44 K	25.08 M
CNN-LSTM-DAE [14]	10.92 M	46.69 M
LMSC-DAE [15]	63.62 K	12.36 M
CPDAE (Full) [16]	2.69 M	355.01 M
TCDAE [20]	0.31 M	60 M
Proposed FMHA-AE	4.28 M	1640 M

**Table 5:** Results of channel expansion on model performance and computational metrics.

Channel Number	Trainable Parameters	MACs	Training Time	PRD(%)	SNR_imp_	RMSE
64	1.82 M	604 M	20,188.35 s	14.94	21.82	0.00549
128	4.28 M	1640 M	32,375.13 s	10.29	25.34	0.0037
256	28.92 M	7305 M	68,500.74 s	10.76	25.49	0.00403

**Table 6:** Performance metrics for different head numbers of FMHA-AE.

Head Numbers of FMHA-AE	2	4	8	16
PRD (%)	11.02	10.29	10.86	10.92
RMSE (Normalized)	0.00412	0.0037	0.00384	0.00402
SNR_imp_ (dB)	24.93	25.34	25.24	25.32

**Table 7:** Performance Metrics for Non-DAE methods in SNR_imp_ across input noise level.

Model	SNR_imp_ (dB)						
Input SNR	−6 dB	0 dB	6 dB	12 dB	18 dB	24 dB	Avg.
FIR	2.94	2.91	2.78	2.31	1.03	−1.57	1.73
IIR	5.26	5.10	4.08	2.38	−0.71	−5.13	1.83
DWT	5.15	5.07	4.20	2.67	0.11	−4.11	2.18
Proposed FMHA-AE	**31.19**	**34.44**	**29.92**	**24.95**	**19.77**	**11.79**	**25.34**

**Table 8:** Performance Metrics for Non-DAE methods in PRD across input noise level.

Model	PRD (%)						
Input SNR	−6 dB	0 dB	6 dB	12 dB	18 dB	24 dB	Avg.
FIR	527.2	267.9	136.4	73.8	40.9	26.8	178.8
IIR	501.9	253.9	134.2	76.1	50.1	39.4	175.9
DWT	508.3	255.6	133.0	73.9	43.7	32.7	174.5
Proposed FMHA-AE	**29.20**	**8.27**	**6.35**	**5.73**	**5.35**	**6.84**	**10.29**

**Table 9:** Comparison of SNR_imp_ between the proposed architecture and other architectures.

Model	SNR_imp_ (dB)						
Input SNR	−6 dB	0 dB	6 dB	12 dB	18 dB	24 dB	Avg.
DNN-DAE [11]	18.83	13.72	8.49	2.62	−2.94	−9.50	5.20
CNN-DAE	18.63	15.11	10.58	5.29	−0.36	−7.08	7.03
FCN-DAE [12]	18.60	14.38	10.80	6.35	2.18	−3.88	8.07
CNN-LSTM-DAE [14]	18.90	15.66	11.24	6.25	0.73	−5.63	7.86
LMSC-DAE [15]	19.30	16.57	12.74	8.78	4.10	0.70	10.37
CPDAE(Full) [16]	23.68	27.75	24.99	21.38	16.92	8.15	20.48
TCDAE [20]	21.27	22.49	19.69	15.35	9.91	4.33	15.51
Proposed FMHA-AE	**31.19**	**34.44**	**29.92**	**24.95**	**19.77**	**11.79**	**25.34**

**Table 10:** Comparison of PRD between the proposed architecture and other architectures.

Model	PRD (%)						
Input SNR	−6 dB	0 dB	6 dB	12 dB	18 dB	24 dB	Avg.
DNN-DAE [11]	88.15	79.13	76.70	73.00	73.65	73.38	77.34
CNN-DAE	89.45	68.19	61.41	54.50	55.38	56.09	64.17
FCN-DAE [12]	93.53	73.29	60.21	49.00	42.01	39.29	59.56
CNN-LSTM-DAE [14]	86.92	65.23	54.68	42.70	47.44	47.38	57.39
LMSC-DAE [15]	86.04	57.88	46.89	37.18	28.68	25.47	47.02
CPDAE(Full) [16]	51.20	18.10	12.81	9.28	8.65	8.66	18.12
TCDAE [20]	69.51	32.30	22.16	17.20	15.29	15.57	28.67
Proposed FMHA-AE	**29.20**	**8.27**	**6.35**	**5.73**	**5.35**	**6.84**	**10.29**

## Data Availability

The ECG datasets used in this study are publicly available. The MIT-BIH Arrhythmia Database (MITDB) and the Noise Stress Test Database (NSTDB) can be accessed from PhysioNet at https://physionet.org. Additional experimental results are available from the corresponding author upon reasonable request.

## References

[1] Addissouky T.A., El Sayed I.E.T., Ali M.M.A., Alubiady M.H.S., Wang Y. (2024). Recent Developments in the Diagnosis, Treatment, and Management of Cardiovascular Diseases Through Artificial Intelligence and Other Innovative Approaches. J. Biomed. Res..

[2] Webster J.G. (1984). Reducing Motion Artifacts and Interference in Biopotential Recording. IEEE Trans. Biomed. Eng..

[3] Huhta J.C., Webster J.G. (1973). 60-Hz Interference in Electrocardiography. IEEE Trans. Biomed. Eng..

[4] Rahman M.Z.U., Shaik R.A., Reddy D.V.R.K. (2012). Efficient and Simplified Adaptive Noise Cancelers for ECG Sensor Based Remote Health Monitoring. IEEE Sens. J..

[5] Sharma I., Mehra R., Singh M. Adaptive filter design for ECG noise reduction using LMS algorithm. Proceedings of the 2015 4th International Conference on Reliability, Infocom Technologies and Optimization: Trends and Future Directions (ICRITO 2015).

[6] Shaddeli R., Yazdanjue N., Ebadollahi S., Saberi M.M., Gill B. Noise Removal from ECG Signals by Adaptive Filter Based on Variable Step Size LMS Using Evolutionary Algorithms. Proceedings of the Canadian Conference on Electrical and Computer Engineering.

[7] Zhang M., Wei G. (2020). An Integrated EMD Adaptive Threshold Denoising Method for Reduction of Noise in ECG. PLoS ONE.

[8] Supraja V., Safiya S. (2013). ECG De-Noising Using Thresholding Based on Wavelet Transforms. Int. J. Eng. Res. Appl..

[9] Yadav S.K., Sinha R., Bora P.K. (2015). Electrocardiogram Signal Denoising Using Non-Local Wavelet Transform Domain Filtering. IET Signal Process..

[10] Vincent P., Larochelle H., Bengio Y., Manzagol P.A. (2008). Extracting and Composing Robust Features with Denoising Autoencoders. Proceedings of the 25th International Conference on Machine Learning.

[11] Xiong P., Wang H., Liu M., Liu X. (2015). Denoising Autoencoder for Eletrocardiogram Signal Enhancement. J. Med. Imaging Health Inf..

[12] Chiang H.T., Hsieh Y.Y., Fu S.W., Hung K.H., Tsao Y., Chien S.Y. (2019). Noise Reduction in ECG Signals Using Fully Convolutional Denoising Autoencoders. IEEE Access.

[13] Hochreiter S., Schmidhuber J. (1997). Long Short-Term Memory. Neural Comput..

[14] Dasan E., Panneerselvam I. (2021). A Novel Dimensionality Reduction Approach for ECG Signal via Convolutional Denoising Autoencoder with LSTM. Biomed. Signal Process Control.

[15] Jhang Y.S., Wang S.T., Sheu M.H., Wang S.H., Lai S.C. (2022). Integration Design of Portable ECG Signal Acquisition With Deep-Learning Based Electrode Motion Artifact Removal on an Embedded System. IEEE Access.

[16] Jhang Y.S., Wang S.T., Sheu M.H., Wang S.H., Lai S.C. (2022). Channel-Wise Average Pooling and 1D Pixel-Shuffle Denoising Autoencoder for Electrode Motion Artifact Removal in ECG. Appl. Sci..

[17] Seo H.C., Yoon G.W., Joo S., Nam G.B. (2022). Multiple Electrocardiogram Generator with Single-Lead Electrocardiogram. Comput. Methods Programs Biomed..

[18] Wang X., Chen B., Zeng M., Wang Y., Liu H., Liu R., Tian L., Lu X. (2022). An ECG Signal Denoising Method Using Conditional Generative Adversarial Net. IEEE J. Biomed. Health Inf..

[19] Vaswani A., Shazeer N., Parmar N., Uszkoreit J., Jones L., Gomez A.N., Kaiser Ł., Polosukhin I. (2017). Attention Is All You Need. Adv. Neural Inf. Process Syst..

[20] Chen M., Li Y., Zhang L., Liu L., Han B., Shi W., Wei S. (2024). Elimination of Random Mixed Noise in ECG Using Convolutional Denoising Autoencoder with Transformer Encoder. IEEE J. Biomed. Health Inf..

[21] Moody G., Mark R. MIT-BIH Arrhythmia Database. https://www.physionet.org/content/mitdb/1.0.0/.

[22] Moody G., Mark R. MIT-BIH Noise Stress Test Database. https://www.physionet.org/content/nstdb/1.0.0/.

[23] He K., Zhang X., Ren S., Sun J. Deep Residual Learning for Image Recognition. Proceedings of the IEEE Computer Society Conference on Computer Vision and Pattern Recognition.

[24] Ibrahem H., Salem A., Kang H.S. (2025). Pixel Shuffling Is All You Need: Spatially Aware Convmixer for Dense Prediction Tasks. Pattern Recognit..

[25] Wang P. Image Denoising Using Deep CGAN with Bi-Skip Connections. Proceedings of the 2019 IEEE/CVF Conference on Computer Vision and Pattern Recognition Workshops (CVPRW).

[26] Park B., Yu S., Jeong J. Densely Connected Hierarchical Network for Image Denoising. Proceedings of the 2019 IEEE/CVF Conference on Computer Vision and Pattern Recognition Workshops (CVPRW).

[27] Dong L.F., Gan Y.Z., Mao X.L., Yang Y.B., Shen C. Learning Deep Representations Using Convolutional Auto-Encoders with Symmetric Skip Connections. Proceedings of the 2018 IEEE International Conference on Acoustics, Speech and Signal Processing (ICASSP).

